# Optical label-free detection of SARS-CoV-2: investigating platform spectroscopic properties for oligonucleotide targeting

**DOI:** 10.1007/s00249-025-01787-3

**Published:** 2025-08-11

**Authors:** Silvia Maria Cristina Rotondi, Paolo Canepa, Silvia Dante, Maurizio Canepa, Ornella Cavalleri

**Affiliations:** 1 OPTMATLAB, Dipartimento Di Fisica, Università Di Genova, Via Dodecaneso 33, 16146 Genoa, Italy; 2 Materials Characterization Facility, Istituto Italiano Di Tecnologia, Via Morego 30, 16163 Genoa, Italy

**Keywords:** SARS-CoV-2, DNA, Self-assembled monolayers, Biosensing, Spectroscopic ellipsometry

## Abstract

We characterized a DNA/gold interface designed for the detection of the SARS-CoV-2 RNA-dependent RNA polymerase/Helicase (RdRp/Hel) sequence. Using broadband spectroscopic ellipsometry (SE) and a difference spectra approach, we monitored molecular modifications at the interface, from probe sequence deposition to the insertion of a molecular spacer and subsequent hybridization with the target. The UV region revealed the characteristic DNA absorption peak around 260 nm, while changes in δΔ in the NIR correlated with increased optical thickness following each deposition step. The optical response was analyzed as a function of target concentration, and the binding affinity curve, derived from δΔ values at 800 nm, was fitted using a first-order Langmuir model, yielding a dissociation constant K_D_ = (70 ± 10) nM, consistent with literature values. Selectivity studies demonstrated that the interface effectively discriminates the SARS-CoV-2 sequence from the SARS-CoV HKU variant, even in a crowded environment. A complementary platform targeting the SARS-CoV HKU sequence confirmed selective detection of HKU over SARS-CoV-2. These findings highlight the potential for parallel detection of different viral sequences.

**Supplementary Information:**

The online version contains supplementary material available at 10.1007/s00249-025-01787-3.

## Introduction

A central challenge in the development of sensitive bio-interfaces lies in the stable and selective immobilization of capture molecules onto the sensor surface. Exploiting the strong affinity between thiol groups and gold, self-assembled monolayers (SAMs) have been widely used to anchor functional moieties to gold substrates while minimizing non-specific adsorption (Ravan et al. 2014; Tewari et al. 2023). Among the various functional groups employed, DNA emerges as a particularly promising and versatile candidate. The DNA/gold interface has been extensively investigated as a biosensing platform (Jones et al. 2015; Xiao et al. 2019; Espinosa et al. 2021; Chen et al. 2022) as it satisfies two essential criteria for effective sensing: (i) the high specificity of DNA, arising not only from Watson–Crick base pairing but also from the selective binding capabilities of DNA aptamers (Meng et al. 2016; Zhang et al. 2020; Feng et al. 2025); and (ii) the chemical stability and ease of surface modification of gold, which make it an ideal substrate for sensor fabrication (Love et al. 2005). Since the late 1990 s (Zhai et al. 1997), DNA-based biosensors have been thoroughly explored for the detection of a wide range of analytes—including toxins, proteins, heavy metals, and environmental pollutants (Zhao et al. 2022; Hua et al. 2022; Samiseresht et al. 2025). Nevertheless, their most prominent and widely adopted application remains the detection of nucleic acids.

In previous studies, we focused on a hybrid DNA/gold platform for the detection of a model 22-mer oligonucleotide sequence (Pinto et al. 2022, 2020, 2019). By integrating optical spectroscopy, atomic force microscopy (AFM), and quartz crystal microbalance (QCM) analysis, we achieved a comprehensive understanding of the hybrid interface. Moreover, broadband spectroscopic ellipsometry enabled us to detect the characteristic UV absorption of DNA self-assembled monolayers (SAMs), suggesting the occurrence of hypochromism in surface-immobilized DNA strands (Pinto et al. 2022). The optical properties of the DNA SAMs were interpreted through a stacked-layer optical model, informed by independent structural data obtained from AFM measurements. Building on this expertise, the present study investigates a label-free sensing platform designed for the specific detection of the RNA-dependent RNA polymerase Helicase (RdRp/Hel) sequence of SARS-CoV-2. This target sequence is highly conserved across all known SARS-CoV-2 variants and has demonstrated the highest analytical sensitivity in RT-PCR assays (Corman et al. 2020), thus qualifying it as an ideal candidate for nucleic acid-based SARS-CoV2 detection. The sensing platform is assembled through a three-step protocol. First, a thiolated single-stranded DNA probe, fully complementary to the RdRp/Hel sequence, is chemisorbed onto the gold surface. In the second step, the DNA-functionalized surface is treated with mercaptohexanol (MCH), an alkanethiol with the same chain length as the DNA thiol linker, which serves as a molecular spacer. MCH enhances the structural organization of the SAM, increases hybridization efficiency (Wong et al. 2005) and minimizes non-specific interactions between nitrogenous bases and the gold surface (Ravan et al. 2014). Finally, the modified surface is exposed to the target sequence to assess the selectivity and efficiency of hybridization.

Following this initial validation, the sensor is evaluated for key performance metrics: reusability, selectivity, and sensitivity. Reusability, previously demonstrated for a model sequence (Pinto et al. 2022), is exploited to regenerate the sensing interface and progressively expose it to lower target concentrations. This approach enables the construction of a calibration curve and the determination of both the dissociation constant K_D_ and the limit of detection (LOD).

To evaluate selectivity, the sensor is challenged with the RdRp/Hel sequence from the earlier SARS-CoV (HKU strain) (Chan et al. 2020). Discrimination between target and non-target sequences is also confirmed under complex conditions in which the non-target was present in excess. Moreover, by designing an analogous sensor using a probe complementary to the HKU sequence, cross-selectivity between the two viral variants was investigated and validated.

## Material and methods

### Materials and chemicals

This study focuses on the 28-mer RNA-dependent RNA-polymerase (RdRp) Helicase sequence of SARS-CoV-2 and SARS-CoV HKU listed in NCBI database (SARS-CoV-2 GenBank accession no. MN908947.3, genome location 16,276–16,303; SARS-CoV HKU GenBank accession no. AY278491, genome location 16,206–16233).

The oligonucleotide sequences, listed from the 5ʹ to the 3ʹend, are reported in the following:

- SARS-CoV-2 probe (HS_p-CoV2): HS-(CH_2_)_6_-GTC TAC GTA TGC AAG CAC CAC ATC TTAA
- SARS-CoV HKU probe (HS_p-HKU): HS-(CH_2_)_6_-GTC T**C**C **TA**A T**A**C A**G**G CAC C**G**C A**A**C **GA**A**G**
- SARS-CoV-2 target (t-CoV2): TTA AGA TGT GGT GCT TGC ATA CGT AGAC
- SARS-CoV HKU target (t-HKU): **C**T**T C**G**T** TG**C** GGT GC**C** TG**T** AT**T A**G**G** AGAC

The SARS-CoV-2 and SARS-CoV HKU sequences differ by 10 nitrogen bases, distributed along the entire strand, highlighted in bold in the above list. Thiolated probe strands are designed to be fully complementary to the corresponding target sequences. Oligonucleotides were purchased from biomers.net GmbH (Ulm, Germany) and used as received.

We note that most of the experiments were conducted using DNA sequences since DNA is more stable and easier to handle than RNA which is prone to degradation by RNases. However, control experiments, carried out using RNA sequences, showed the same behavior.

Tris[hydroxymethyl]amino-methane (Tris base), ethylenediaminetetraacetic acid (EDTA), Sodium chloride (NaCl), Sodium Hydroxide (NaOH) and 6-Mercapto-1-hexanol (HS–(CH_2_)_6_–OH, MCH) were purchased from SigmaAldrich (St. Louis, MO, USA). Sulfuric acid (H_2_SO_4_) and 30% hydrogen peroxide (H_2_O_2_) were purchased from Carlo Erba (Val de Reuil, France). Ultrapure water, MilliQ water from Millipore (resistivity 18.2 MΩ·cm), was used for the preparation of all the solutions.

The experiments were performed in TE buffer (10 mM Tris,1 mM EDTA, 1 M NaCl, pH adjusted to 7.2 using HCl (Fluka, Buchs, Switzerland)).

Flat gold substrates (200 nm of gold on glass, with 2 nm of Chromium as an adhesion layer), purchased from Arrandee (Werther/Westfalen, Germany), were used for SE measurements.

### Sample preparation

Arrandee substrates were cleaned in piranha solution (4:1 H_2_SO_4_:30%H_2_O_2_) for 3 min, then rinsed in MilliQ water and dried under a nitrogen stream (CAUTION, Piranha should be handled with extreme care: it is extremely oxidizing, reacts violently with organics and should only be stored in loosely tightened containers to avoid pressure buildup).

**Fig. 1:**
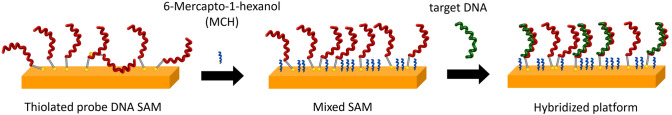
Representative scheme of the sensing platform. After chemisorption of the thiolated probe DNA, the molecular spacer MCH is inserted. The platform results in a mixed pDNA/MCH SAM and is ready for hybridization following the exposure to target DNA

Samples were prepared according to the following deposition protocol: 3 h immersion in a 1 μM solution of thiolated DNA, rinsing in TE buffer, 1 h immersion in 5 μM solution of MCH, rinsing in TE Buffer, 1 h incubation in the target sequence solution, and rinsing in TE buffer. A representative scheme of the system is reported in Fig. 1. For the regeneration of the platform, samples were immersed in a 1 M solution of NaOH for 3 min and then rinsed in TE buffer.

### Spectroscopic ellipsometry

Spectroscopic Ellipsometry (SE) measurements were performed using a rotating compensator instrument (M-2000, J.A. Woollam Co., Lincoln, NE, USA, 245–1700 nm) equipped with a 75 W Xe lamp. Spectra have been collected in situ using a commercial liquid cell (J.A. Woollam Co., 0.5 mL).

All static spectra were measured in TE buffer after completion of each molecular deposition step, as detailed above. To emphasize the contribution of the ultrathin organic layer, we analysed difference spectra, obtained as the difference between the spectra acquired after the film deposition and the spectra measured on the clean gold substrate prior to molecular deposition, both acquired in TE buffer.

## Results and discussion

### SE static spectra

**Fig. 2:**
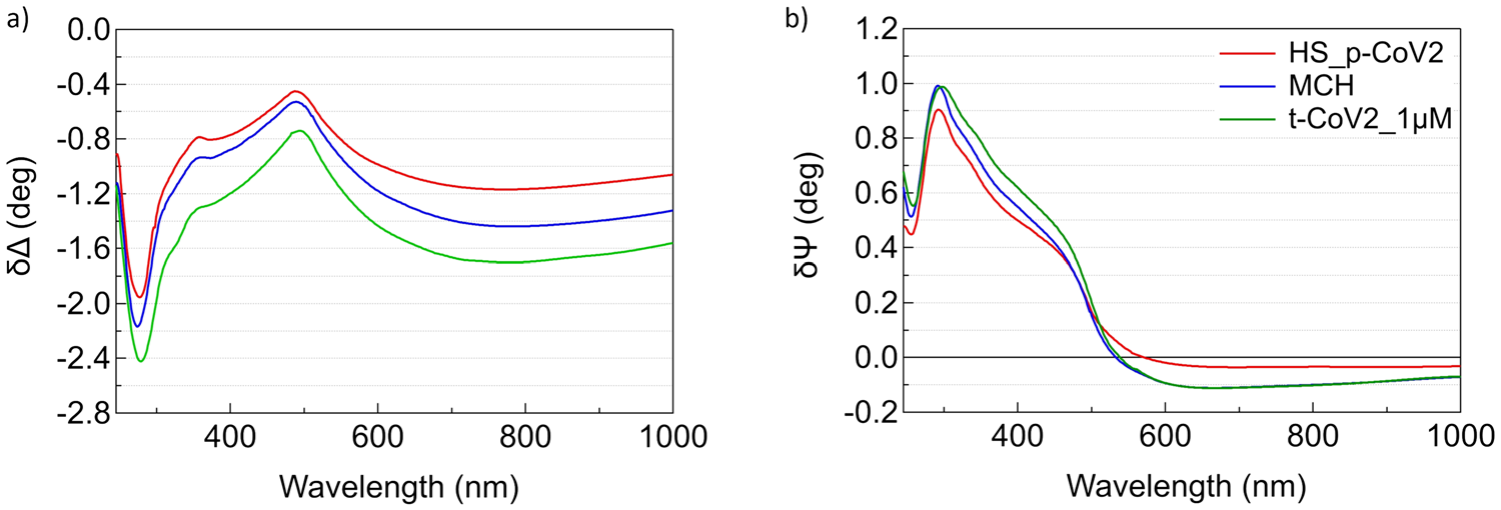
**a** δΔ and **b** δψ static difference spectra referred to cleaned gold obtained after deposition of the thiolated probe DNA HS_p-CoV2 (red spectra), MCH (blue spectra), and after hybridization with a 1 μM solution of t-CoV2 (green spectra). We note that above 500 nm blue and green δψ curves are superimposed, as expected since target DNA does not interact directly with gold and, therefore, does not produce changes at the molecule/gold interface

Broadband SE static measurements were performed to monitor the assembly of the DNA sensing interface, i.e. the deposition of the thiolated probe DNA, the insertion of the molecular spacer, and the hybridization with the target sequence, the RdRp/Helicase of Sars-CoV2. Static measurements were performed in TE buffer after reaching steady state conditions as evidenced by dynamic measurements (see SI, Fig.S1). To highlight the SAM contribution, for each deposition step, we calculated difference spectra referenced to the cleaned gold substrate. δΔ and δψ difference spectra obtained after the deposition of the thiolated strand (red curve), the MCH spacer (blue curve) and the hybridization with the target sequence (green curves) are reported in Fig. 2a and b, respectively.

Each molecular adsorption step produces a quasi-rigid downward shift of δΔ as well as an increase of δψ below 500 nm. We discussed these spectral changes in detail in a previous paper on 22-mer SAMs, which behave similarly to the 28-mer SAMs from a SARS-CoV-2 sequence examined here, showing that these spectral changes are associated to an increase of the film optical thickness (Pinto et al. 2022).

It is worth noticing that MCH is shorter than the DNA thiolated probe, comparable in length to the thiolated linker of the probe DNA. The increase of optical thickness following the insertion of the molecular spacer can then be ascribed to a reorganization of the probe DNA film: the MCH thiol group displaces the weak adsorptive contacts between DNA strands and gold, leaving the probes tethered primarily through the thiol end groups. Probe DNA therefore swells and extends further into solution (Steel et al. 2000; Lao et al. 2005). After the exposure to the target DNA, the increase in optical thickness can be reasonably attributed to the reorganization/stretching of DNA strands upon hybridization.

The negative values of δψ above 500 nm point to a strong interaction between molecules and substrate, as previously reported for thiolated (Prato et al. 2008; Pinto et al. 2022) and selenolated (Canepa et al. 2013) SAMs on gold. Chemisorption of HS-p-CoV2 and MCH on gold, through the thiol group, results in increasingly negative δψ values, while no further decrease is observed after hybridization, since t-CoV2 does not interact directly with gold.

In passing, we note that the dip around 260 nm of both δΔ and δψ, not observed in difference spectra of ultrathin transparent SAMs (Prato et al. 2008; Solano et al. 2015), is the spectral signature of the DNA UV absorption, detected for immobilized DNA at the single monolayer level (Pinto et al. 2022). This confirms the high sensitivity of the difference spectra analysis in highlighting UV–Vis molecular absorption down to the monolayer, as previously reported for monolayers of Cytochrome *c* on gold (Toccafondi et al. 2011).

### Platform response at different target concentrations

**Fig. 3:**
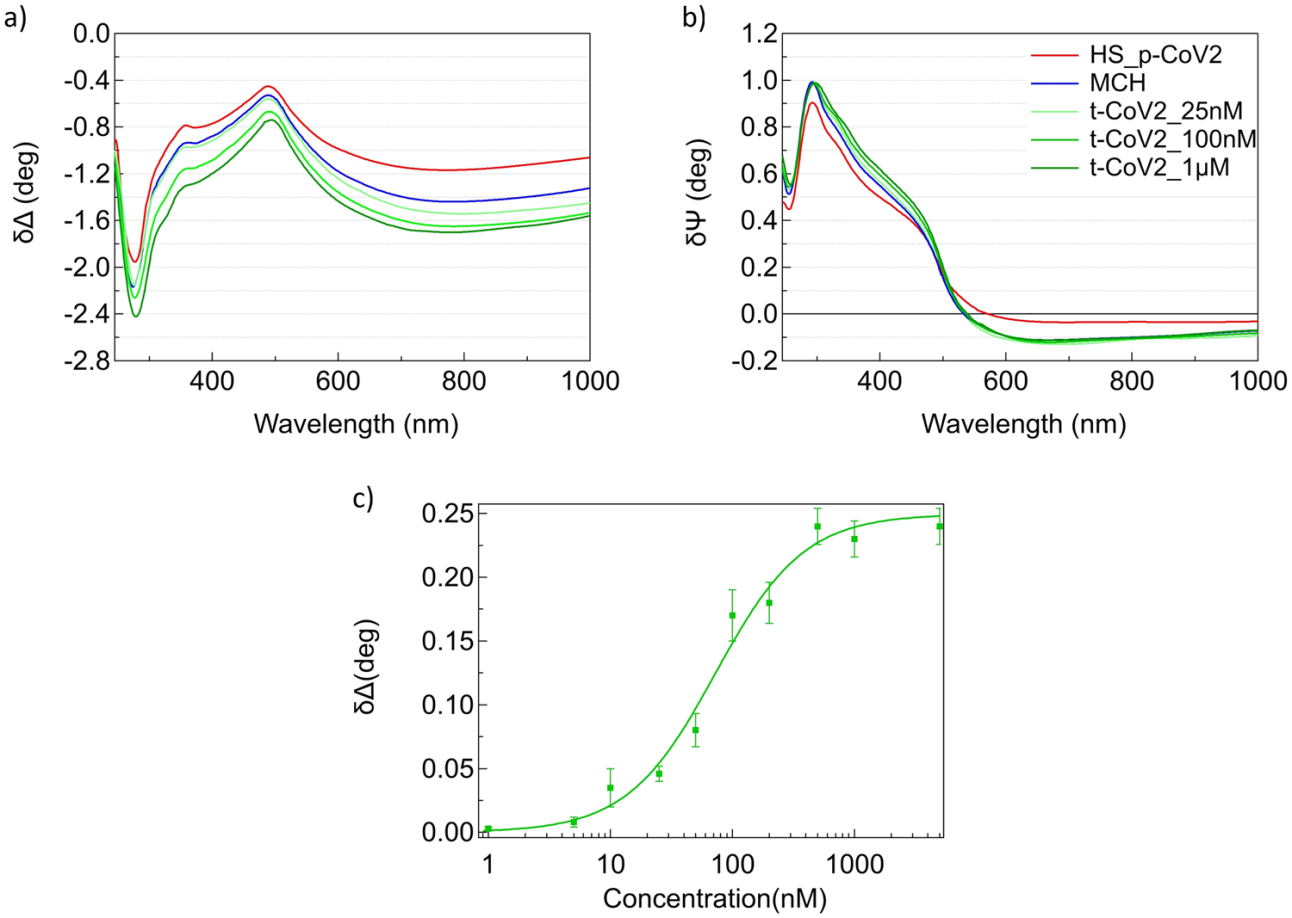
**a** δΔ and **b** δψ difference spectra referred to the gold substrate after the deposition of thiolated DNA HS_p-CoV2 (red curve), MCH (blue curve) and hybridization with different concentrations of t-CoV2, 25 nM, 100 nM and 1 μM (from light to dark green curves). **c** δΔ values calculated as the difference between t-CoV2 and MCH Δ values at 800 nm at different t-CoV2 concentrations. Hill equation is adapted to experimental data. Error bars are the semidifference of δΔ@800 nM between the maximum and minimum values at each concentration

Once assessed the main features of the SE difference spectra of the hybrid DNA/gold interface, we characterized the sensing platform by studying the optical response of the interface to hybridization as a function of the target concentration. Representative δΔ and δψ spectra obtained after exposure of the mixed HS-p-CoV2/MCH SAM to 25 nM, 100 nM and 1 µM solutions of t-CoV2 for 1 h are reported in Fig. 3a, b. As expected, increasing the target concentration produces increasingly larger downward shifts of δΔ and increases of δψ around 400 nm, while no δψ changes occur in the NIR region. Since the Δ decrease in the NIR is larger than the ψ increase around 400 nm (Fig. 3a, b), we focussed on the analysis of δΔ at 800 nm to quantitatively measure the interface optical response to the target sequence. Exploiting the regeneration of the sensing platform via alkaline rinsing (see SI, Fig.S2), for each sample we performed cycles of hybridization/dehybridization experiments at different target concentrations. To standardize the procedure, a fixed incubation time of 1 h was chosen for all target concentrations.

The resulting hybridization binding curve is shown in Fig. 3c. δΔ values calculated as the difference between Δ@880 nm measured after hybridization and Δ@880 nm measured on the HS-p-CoV2/MCH SAM were plotted versus t-CoV2 concentration (ranging from 1 nM to 5 µM). The binding affinity curve characterizes the sensing response of the platform. At a concentration larger than 500 nM, the system reaches saturation, while the Limit of Detection (LOD) concentration is around 16 nM (see SI3, Fig.S3). Langmuir-Hill equation was fitted to the sigmoidal data distribution (Finlay et al. 2020) obtaining a dissociation constant K_D_ = (70 ± 10) nM, corresponding to an association constant K_A_ = (1.43 ± 0.16)·10^7^ M^−1^. These values are in good agreement with results reported in literature for similar systems (Kambhampati et al. 2001; Nelson et al. 2001; Peterson et al. 2002; Li et al. 2007; White et al. 2015; Botti et al. 2023). In a SPR imaging study, an association constant of 1.8 10^7^ M^−1^ was obtained for 18-mer DNA strands (Nelson et al. 2001), while a K_A_ of 6 10^7^ M^−1^ was obtained for 25-mer DNA strands (Peterson et al. 2002). Nielsen and co-workers (Kambhampati et al. 2001) report K_A_ = 4 10^7^ M^−1^ for 15-mers attached to a dextran matrix.

### SE selectivity

Once verified the recognition capability for the target sequence, we assessed the selectivity of the system for the RdRp-Helicase sequence of SARS-CoV-2 by exposing the platform to the same sequence of another virus of the coronavirus family, namely the HKU SARS-CoV, which displays 10 mismatches compared to the corresponding SARS-CoV-2 sequence. The mixed HS-p-CoV2/MCH SAM was exposed to the t-HKU sequence following the same incubation protocol used for the experiments reported in Fig. 2a, b (1 h incubation in 1 µM t-HKU solution). The resulting δΔ and δψ static spectra (Fig.S4) show no changes upon exposure to t-HKU pointing to a good selectivity of the sensing platform.

**Fig. 4:**
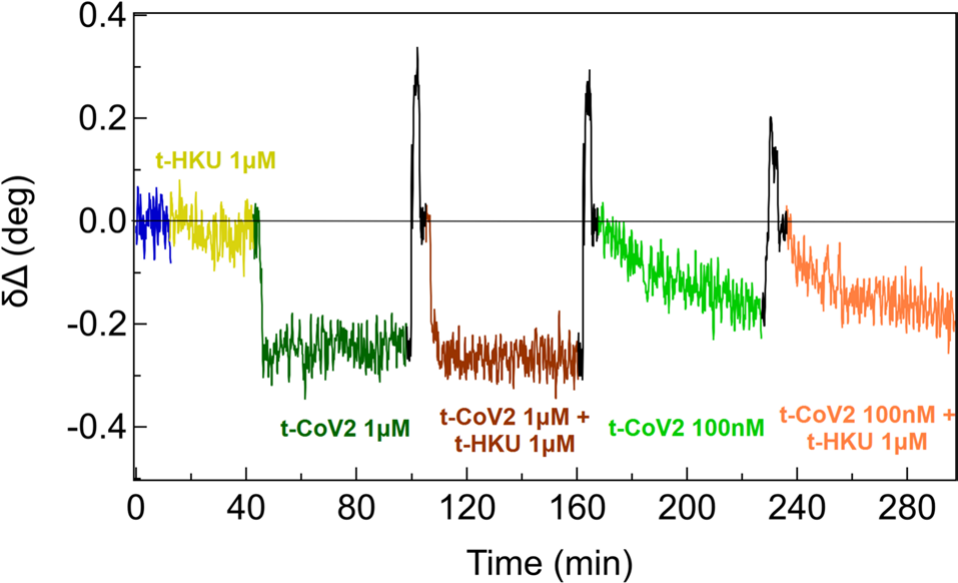
Dynamics of Δδ@800 nm starting from the MCH level (blue curve), exposing the system to a 1 μM solution of the non-target sequence t-HKU (yellow curve), a 1 μM solution of the target sequence t-CoV2 (dark green curve), a mixture of t-CoV2 and t-HKU both at 1 μM (brown curve), a 100 nM solution of t-CoV2 (light green curve), and a mixture of t-CoV2 100 nM and t-HKU at 1 μM (orange curve). Incubation in NaOH 1 M for platform regeneration is reported in black. A horizontal curve highlights the zero level to which the system goes back after every dehybridization

To further test the system’s capability to discriminate between target and non-target sequences in crowded environments, the sensing platform was exposed to a mixture of t-CoV2 and t-HKU sequences. The resulting static difference spectra (not shown) indicate that the competitive presence of the non-target sequence does not affect the platform optical response. This is further evidenced by dynamic measurements as reported in Fig. 4. δΔ values measured at 800 nm during incubation in a “pure” solution of t-CoV2 at different concentrations (1 μM and 100 nM, dark and light green curves) and in a mixture of t-CoV2 and t-HKU sequences are reported in Fig. 4. In the mixture, t-CoV2 concentration was changed from 1 μM to 100 nM (dark and light brown curves), while t-HKU concentration was kept constant at 1 μM. This leads to the exposure of the platform to a solution that contains the non-target sequence at a concentration up to 10 times the concentration of the target sequence. Nonetheless, both in this case and for the equal concentration of target and non-target, the system response was similar to the response of a pure target solution. This points out that the presence of non-target sequences does not affect the detection of the target sequence, even when the former is in excess. Dynamic measurements provide further information on the kinetics of hybridization; as exemplified by the comparison of hybridization measurements in 100 nM and 1 µM target concentration (Fig. 4), slower hybridization rates are observed at lower target concentrations, as reasonably expected. This aspect will be further investigated in future studies.

### HKU platform

**Fig. 5:**
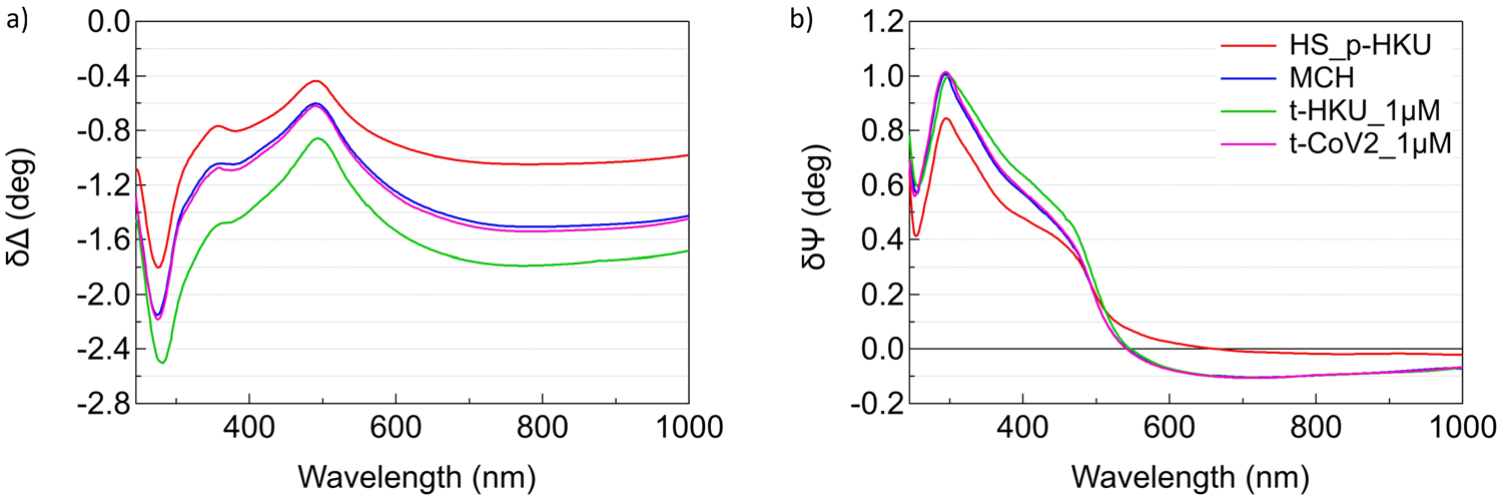
Difference spectra in **a** δΔ and in **b** δψ referred to the gold substrate after the deposition of thiolated DNA HS_p-HKU (red curve), MCH (blue curve), hybridization with t-HKU 1 μM (green curve) and after exposition to non-target sequence t-CoV2 1 μM (pink curve)

To further characterize the selectivity of the sensing platform, we performed complementary measurements by immobilizing the probe sequence for HKU (HS_p-HKU) on the surface and exposing it to its target sequence t-HKU and to the SARS-CoV-2 target. The resulting static difference spectra are reported in Fig. 5. The target sequence hybridizes with the immobilized probe resulting in a vertical quasi-rigid shift in δΔ and an increase around 400 nm in δψ (green curve, obtained upon exposure to 1 μM t-HKU solution), while the exposure to t-CoV2 1 μM solution does not produce significant changes in the difference spectra (pink curve).

The possibility to implement the sensor platform for different target sequences, while maintaining selectivity even for sequences with a limited number of mismatches, supports its potential for multiplex targeting of different sequences.

It is interesting to note some differences between the static spectra obtained on the CoV-2 and HKU platforms. Focusing on the NIR region, where the decrease in Δ is related to the increase of the film optical thickness, the representative spectra of the CoV-2 platform (Fig. 2a) show a larger decrease in Δ after the deposition of probe DNA compared to the HKU platform. On the other hand, the decrease in Δ following the deposition of MCH is greater in the HKU case than in the CoV-2 case.

This observation can be tentatively ascribed to the different propensity of the probe sequences of CoV-2 and HKU to form loops. In fact, DNA secondary structure predictions (see SI5) suggest that the HKU probe a greater tendency to form loops. As a consequence, a larger molecular footprint is expected for the HKU probe, potentially leading to a lower surface density compared to the CoV-2 probe. If fewer p-HKU molecules occupy binding sites on the gold surface, more sites would remain available for MCH chemisorption, leading to a more extensive reorganization of the self-assembled monolayer (SAM) upon MCH insertion. This could have two main effects. Regarding Δ, it may result in a slightly lower optical thickness for HS-p-HKU and a more pronounced decrease in Δ for the mixed film in the HKU platform. Regarding δΨ, above 500 nm, the p-HKU alone appears to exhibit slightly negative values, less negative than for the p-CoV2, while MCH chemisorption seems to lead to a noticeable decrease in δΨ. Further investigations, combining SE with AFM and QCM-D, will be necessary to validate this analysis.

## Conclusions

We characterized a DNA/gold interface specifically designed for the targeting of a SARS-CoV-2 sequence, the RdRp/Helicase sequence. To this end, we leveraged previous analysis of a hybrid DNA/gold interface assembled using a model 22-mer nucleotide sequence.

The target detection is operated through the acquisition of broadband SE spectra, which are analysed through a difference spectra approach to enhance the contribution of the ultrathin organic layer. Broadband difference spectra from UV to NIR allowed to monitor the step-by-step changes occurring at the interface from the deposition of the probe sequence to the insertion of the molecular spacer, the two steps that define the sensing platform, and finally to the exposure to the target sequence, resulting in hybridization. The UV region shows the fingerprint of the DNA absorption around 260 nm, while the decrease of δΔ in the NIR region and the increase of δψ around 400 nm are related to the increase of the film optical thickness following each deposition step. For each wavelength, Δ and ψ dynamic scans were acquired to monitor molecular deposition in real time.

The interface optical response was measured as a function of the target concentration. The binding affinity curve derived from the δΔ values at 800 nm measured after hybridization as a function of the target concentration was analysed with a first-order Langmuir model, retrieving a dissociation constant K_D_ = (70 ± 10) nM, in agreement with the results reported in literature for similar systems.

Static and dynamic measurements proved that the interface selectively targets the SARS-CoV-2 sequence vs the same sequence of SARS-CoV HKU, a previous coronavirus. The detection efficiency is preserved in a crowded environment containing an excess of HKU sequence.

Similar experiments, conducted on a SARS-CoV HKU platform, demonstrated the selective targeting of t-CoV HKU vs t-CoV2. The possibility to target different sequences paves the way for the parallel detection of different oligonucleotides. Indeed, future studies are planned to integrate AFM nanolithography with SE imaging to enable parallel optical detection of multiple sequences by interrogating arrays of AFM-grafted platforms functionalized with different p-DNAs. The parallel detection of viral sequences that are highly conserved across different SARS-CoV2 variants, such as the RdRp-helicase region, and sequences that are mutated in the different variants, such as sequences related to the receptor binding domain of the spike protein, could enable the differential identification of the distinct SARS-CoV2 variants.

The ability to target different sequences by simply modifying the probe design makes the platform very versatile and highly adaptable for multiplex detection. This feature is particularly useful for applications such as screening viral sequences in infectious disease diagnostics or identifying miRNA sequences dysregulated in pathological conditions.

## Supplementary Information

Below is the link to the electronic supplementary material.

Supplementary file1 (PDF 649 kb)

## Data Availability

The data presented in this study are available from the corresponding authors upon request.
