# Supplementary material for: Optical label-free detection of SARS-CoV-2: investigating platform spectroscopic properties for oligonucleotide targeting
Source: Eur Biophys J. 2025 Aug 11;55(2):209–16. doi: 10.1007/s00249-025-01787-3 (PMC13109271; doi:10.1007/s00249-025-01787-3)
Supplement: Supplementary file 1 — Supplementary file1 (PDF 649 kb) [file 249_2025_1787_MOESM1_ESM.pdf]

Optical Label-Free Detection of SARS-CoV-2: investigating platform spectroscopic properties for oligonucleotide targeting

Silvia Maria Cristina Rotondi[1], Paolo Canepa[1*], Silvia Dante[2], Maurizio Canepa[1,3], Ornella Cavalleri[1*]

[1]Dipartimento di Fisica and OPTMATLAB, Università di Genova, Via Dodecaneso 33, 16146, Genova, Italy;

[2]Materials Characterization Facility, Istituto Italiano di Tecnologia, Via Morego 30, 16163, Genova, Italy

[3]INFN, Sezione di Genova, Via Dodecaneso 33, 16146 Genova, Italy

* corresponding authors: paolocanepa@unige.it, ornella.cavalleri@unige.it

Supporting Informations

SI1 SE Dynamics

Dynamic ellipsometry measurements allow for the real time monitoring of molecular deposition. Monitoring Δ at 800 nm provides information on the optical thickness of the film (see Fig.S1). As was observed in static spectra, at each deposition step the optical thickness increase. After each molecular injection, Δ decreases until steady state conditions are reached. The absolute value of changes at 800 is very small, that suggests a limited optical contrast between the film and the ambient, throughout the whole experiment.

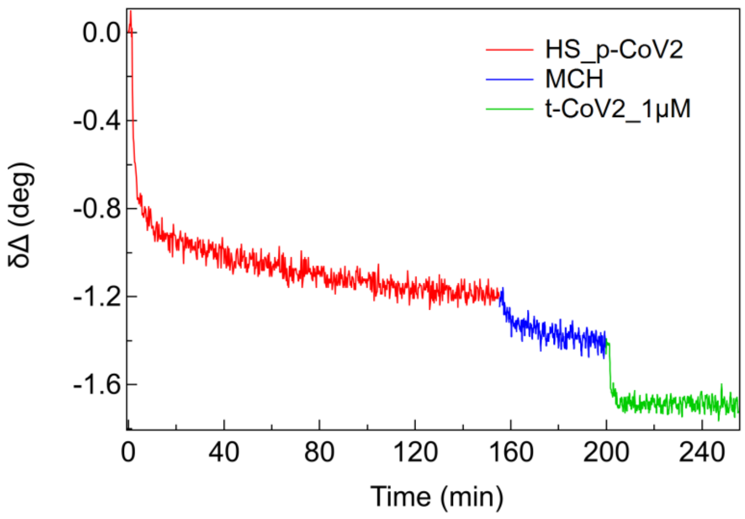

Fig.S1 Ellipsometric dynamic in δΔ at 800nm for the three deposition steps, the thiolated probe HS_pCoV2 (red curve), the molecular spacer MCH (blue curve), and the target sequence t-CoV2 1μM (green curve)

SI2 Platform regeneration

After the first hybridization it is possible to regenerate the sensing platform by exposing it to a 1M solution of NaOH. The OH^- groups of the alkaline solution will interfere with the hydrogen bonds between the complementary bases, causing the denaturation of the double helix. After keeping the system in NaOH for 3 minutes, the system is rinsed thoroughly in TE buffer to remove all the complementary strands and the NaOH residues. The system therefore goes back to the MCH level, as depicted by the black curve both for δΔ and δψ in Fig.S2. By re-exposing the system to a 1μM solution of t-CoV2 the system response is the same of the first hybridization. This testifies the regeneration properties of the platform, which was proved to maintain the same hybridization efficiency after multiple cycles (Pinto et al., 2022).

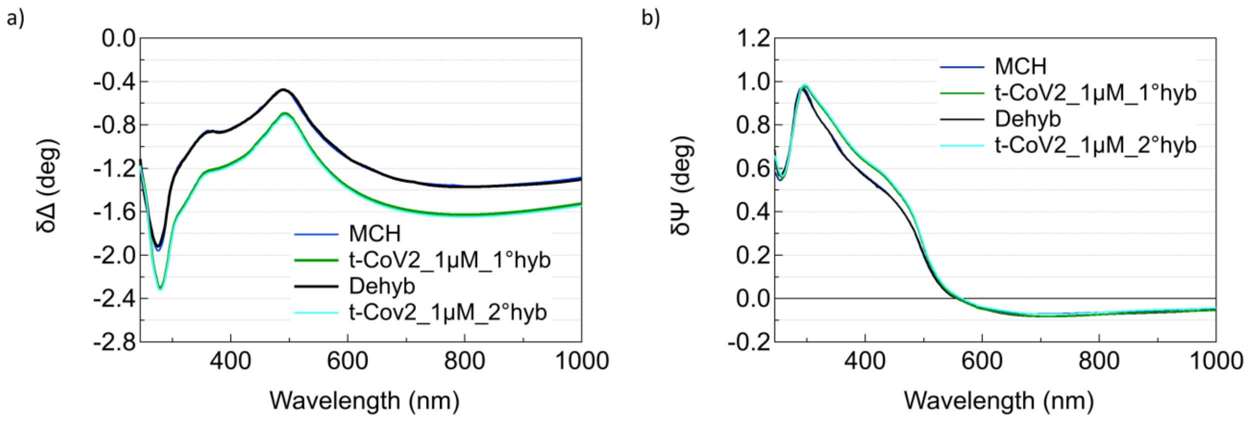

Fig.S2 a) δΔ and b) δψ difference spectra referred to the gold substrate after the deposition of MCH (blue curve) and first exposition to target sequence t-CoV2 1μM (green curve). Exposing the system to a 1M solution of NaOH the system goes back to the MCH level (black curve), and upon re-exposition to a 1μM target sequence the light blue curve overlaps the previous hybridization curve

SI3 LOD determination

The system's Limit of Detection (LOD) can be determined from the calibration curve. As previously established in the literature (Cennamo et al., 2022; De Andrade Silva et al., 2024), the LOD is calculated using the following equation:

$$LOD = \frac{3 \cdot \sigma}{S} \qquad \text{(Eq. S1)}$$

where σ is the standard deviation of the response and S in the slope of the calibration curve. The slope is obtained by fitting a linear regression to the data in the low-concentration region, as reported in Fig3S. The slope is found to be $(1.6 \pm 0.1) \cdot 10^{-3}$deg/nM. The error σ was estimated using the residual standard deviation (RSD) of the regression line. Applying Eq.S1 a LOD of 16 nM was calculated.

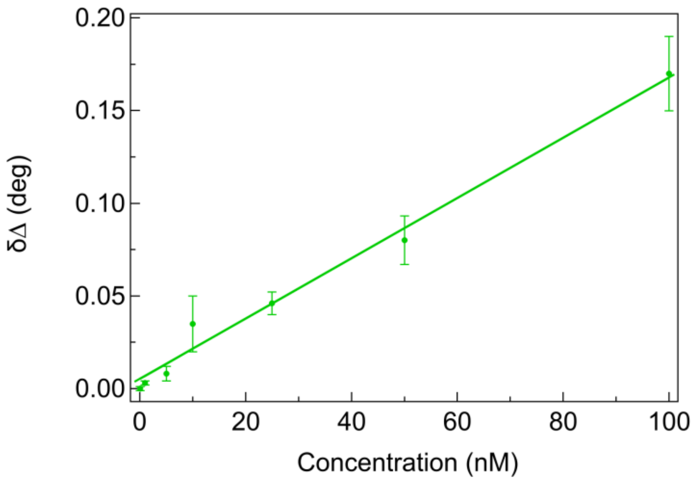

Fig.S3 *Linear equation fitted to δΔ variation at 800nm after the hybridization with different concentrations of the target sequence.*

SI4 Platform selectivity

Difference spectra obtained after exposure to 1μM t-HKU in δΔ and δψ are reported in Fig.S4a,b. After the exposition to the non-target sequence, δΔ and δψ spectra are almost superimposed to the ones measured before exposure to the t-HKU sequence (i.e. at the MCH level), providing evidence that the system can discriminate between Sars-CoV2 and previous coronavirus (SARS-CoV HKU).

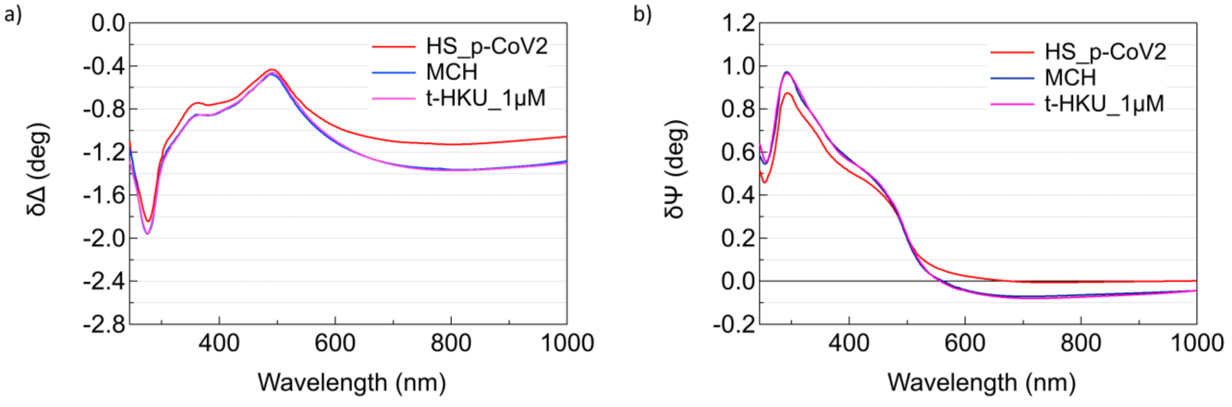

Fig.S4 δΔ a) and δψ b) difference spectra referred to the gold substrate after the deposition of thiolated DNA HS_p-CoV2(red curve), MCH (blue curve), and exposition to non-target sequence t-HKU 1μM (pink curve)

SI5 DNA secondary structure prediction

Secondary structure predictions, obtained through SnapGene Software (www.snapgene.com), are reported in FigS5, both for the probe and target sequence. While both Sars-CoV2 related sequences show no internal loops, HKU related sequences have a good probability to form a loop stabilized by three base pairings.

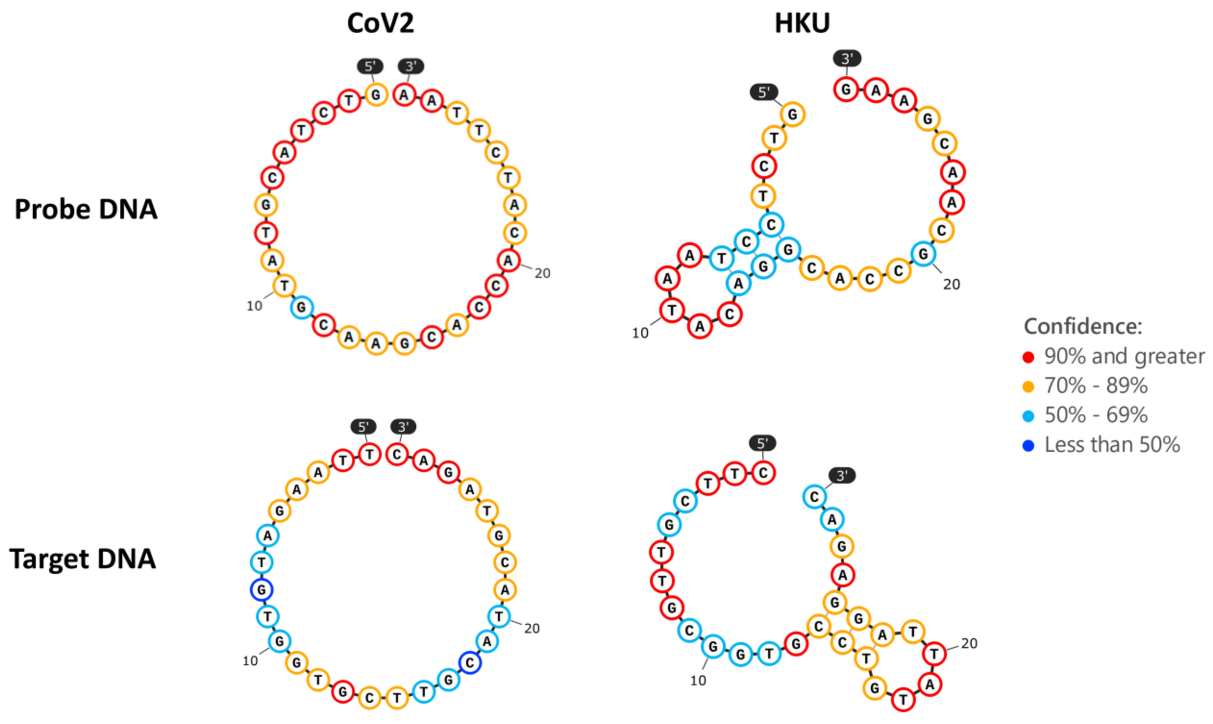

Fig.S5 Prediction of the secondary structure of SARS CoV2 and HKU, probe and target sequences. Reported data were obtained through SnapGene Software (www.snapgene.com)

References

Cennamo, N., Arcadio, F., Seggio, M., Maniglio, D., Zeni, L., Bossi, A.M., 2022. Spoon-shaped polymer waveguides to excite multiple plasmonic phenomena: A multisensor based on antibody and molecularly imprinted nanoparticles to detect albumin concentrations over eight orders of magnitude. Biosensors and Bioelectronics 217, 114707. https://doi.org/10.1016/j.bios.2022.114707

De Andrade Silva, T., Arcadio, F., Zeni, L., Martins, R., De Oliveira, J.P., Marques, C., Cennamo, N., 2024. Plasmonic immunosensors based on spoon-shaped waveguides for fast and on-site ultra-low detection of ochratoxin A in coffee samples. Talanta 271, 125648. https://doi.org/10.1016/j.talanta.2024.125648

Pinto, G., Dante, S., Rotondi, S.M.C., Canepa, P., Cavalleri, O., Canepa, M., 2022. Spectroscopic Ellipsometry Investigation of a Sensing Functional Interface: DNA SAMs Hybridization. Adv Materials Inter 9, 2200364. https://doi.org/10.1002/admi.202200364
